# Are letter identity and letter order encoding functionally separable? Evidence from masked priming with visual similarity and letter transpositions

**DOI:** 10.3758/s13423-026-02989-2

**Published:** 2026-08-26

**Authors:** Ana Marcet, Raquel Ros-García, Pablo Gómez, Melanie Labusch, Manuel Perea

**Affiliations:** 1 Departamento de Didáctica de la Lengua y la Literatura, Universitat de València, Valencia, Spain; 2 Psychology Department, Skidmore College, Saratoga Springs, NY USA; 3 Health Research Institute, La Fe, Valencia, Spain; 4 Departamento de Metodología and ERI-Lectura, Universitat de València, Av. Blasco Ibáñez, 21, 46010 Valencia, Spain; 5 Centro de Investigación Nebrija en Cognición, Universidad Nebrija, Madrid, Spain

**Keywords:** Masked priming, Orthographic processing, Visual word recognition, Transposed-letter priming, Letter identity, Letter position coding

## Abstract

Skilled word reading requires the rapid encoding of both letter identities and letter order. Whereas many models of visual word recognition compute letter order over abstract, case-invariant letter identities, other accounts allow uncertainty about letter identity and letter position to remain jointly constrained during early orthographic processing. We pit these competing claims against each other in a series of masked priming experiments. As a manipulation check for the use of uppercase primes and lowercase targets, Experiment 1 (substitution priming) confirmed a visual-similarity priming effect: Priming was larger for visually similar than visually dissimilar substitution primes. The key tests were Experiments 2–3, which contrasted transposed-letter with replacement-letter control primes in lexical decision and same–different tasks, while varying the visual similarity of the transposed pair. Transposed-letter priming was sizable across tasks and, critically, its magnitude was comparable for visually similar and visually dissimilar transpositions. Taken together, our findings favor a functional dissociation between letter-identity and letter-order encoding. These data place quantitative constraints on accounts in which early visual-form uncertainty is expected to propagate directly into uncertainty about relative letter position in masked priming, and they are more naturally accommodated by models in which letter order is encoded over abstract orthographic representations.

Skilled readers can read sentences like “*IN COURT, WHEN THE WITNESS IDENTIFIED THE SUSPECT, SHE POINTED AT HIM WITH HER FINCER, AS INSTRUCTED BY THE JUGDE*” with only a minimal reading cost, despite the visually confusable G→C substitution in *FINGER*→*FINCER* and the transposition of D and G in *JUDGE*→*JUGDE* (Rayner & Kaiser, 1975; Rayner et al., 2006). This illustrates a hallmark of visual word recognition: The orthographic system tolerates minor distortions in *what* letters are present (letter identities) and *where* they appear (relative letter position; Grainger, 2018). The central question is how, when, and whether letter-identity (the “*what*”) and relative-position (the “*where*”) information interact during visual word recognition.

A widespread assumption in models of orthographic processing is that the encoding of letter order that supports visual word recognition is computed primarily over relatively abstract, case-invariant letter identities (the *Identity-then-order* hypothesis). This assumption is made explicit in the Local Combination Detector (LCD) model (Dehaene et al., 2005), in which visual features activate letter fragments, which then provide input to ordered multi-letter representations with limited positional flexibility (Dehaene & Cohen, 2007). In this view, letter-order coding depends on representations that have already undergone substantial abstraction from visual form, even if activation may cascade continuously between levels (see McClelland, 1979). Critically, however, the transition from visual features to abstract letter identities is complex, where the encoding of letter identity may emerge from a dynamically constrained mapping among feature-level evidence, orthographic structure, and lexical knowledge (see Balota et al., 2006). Most current computational models of visual word recognition adopt a similar approach, in which visual features map onto abstract letter identities, and order is derived from those identities using a flexible code. For example, the spatial coding model represents order as graded spatial patterns over abstract letter units (Davis, 2010; see also Gomez et al., 2008), whereas open-bigram accounts posit location-specific letter detectors feeding order-sensitive bigram units (Grainger & van Heuven, 2003; Grainger & Ziegler, 2011; Whitney, 2001). Although these models differ in the format of the order code (spatial gradients vs. bigrams), they converge on a common assumption: that the encoding of order information operates on letter-identity representations that are already largely abstracted from visual form. The noisy-channel model (Norris & Kinoshita, 2012) differs in mechanism and level of explanation because, rather than positing a specialized orthographic order code, it treats visual word recognition as Bayesian decoding of probabilistic “letter objects” whose identity, order, and even presence are jointly uncertain and must be resolved in parallel as evidence accumulates.

These contrasting views nonetheless crystallize into a critical issue: whether letter-order coding operates on abstract letter-identity representations or whether early form-level and order-level uncertainty are jointly resolved, such that visual confusability increases uncertainty in identity–position binding. Recent neurocomputational studies (e.g., Agrawal & Dehaene, 2024; Yin et al., 2023) provide a useful backdrop by characterizing a transformation along the ventral stream from retinotopic letter coding to more word-centered (ordinal) position coding. Agrawal and Dehaene (2024) implemented convolutional networks that can convert retinotopic letter information into an ordinal letter-position code via intermediate detectors anchored to word boundaries. Converging fMRI/MEG evidence has shown that retinotopic encoding dominates early latencies (approximately 60–200 ms) and that ordinal coding becomes more prominent later (approximately 220–450 ms), while retinotopic information remains present throughout, consistent with mixed coding rather than a clean handoff (Agrawal & Dehaene, 2025).

Masked priming offers a particularly direct window onto these earliest moments of orthographic processing (Forster & Davis, 1984; Grainger, 2008). Two robust phenomena emerging from this paradigm are particularly relevant for evaluating *the potential dynamic interplay between letter identity and letter order*: visual-similarity priming and transposed-letter priming.

Visual-similarity priming refers to the finding that a briefly presented prime in which one letter is replaced by a visually similar character facilitates target processing more than a prime replaced by a visually dissimilar character. This phenomenon was first demonstrated with letter-like digits and symbols (*M4T3R1AL*–*MATERIAL* faster than *M6T8R26L*–*MATERIAL*; Perea et al., 2008; see also Kinoshita et al., 2014; Molinaro et al., 2010). Critically, it also occurs with letters: responses to target words are faster when the prime has been created by replacing an interior letter with a visually similar letter (e.g., *obiect* for *OBJECT*, *i* replaces *j*) than when the letter is visually dissimilar (e.g., *obaect*; *a* replaces *j*; Marcet & Perea, 2017, 2018; see Gutiérrez-Sigut et al., 2019, for ERP evidence; Bae et al., 2024, for evidence in Korean Hangul; Lally & Rastle, 2022, for evidence in perceptual-identification tasks).

Transposed-letter priming is the finding that a briefly presented prime in which two letters are transposed (e.g., *jugde*–JUDGE) facilitates target processing more than a control prime in which both of these letters are replaced (e.g., *jupte*–JUDGE; Perea & Lupker, 2003, 2004). Notably, this priming effect is sizable for both adjacent and nonadjacent transpositions (with one intervening letter; e.g., *cholocate*–CHOCOLATE vs. *chotonate*–CHOCOLATE; Perea & Lupker, 2004), indicating substantial flexibility in order coding during word recognition.

These two phenomena have usually been studied in isolation. Transposed-letter priming is examined while ignoring visual similarity constant, and visual similarity is examined while holding order constant. This leaves open a critical question: is letter-order tolerance computed over abstract letter identities (as per the *identity-then-order* assumption), or is it strengthened when the transposed letters are visually similar—because identity uncertainty makes “*which* letter was *where*” harder to resolve?

One relevant clue comes from unprimed same–different matching. Massol and Grainger (2024) reported that visual-similarity effects generalize across stimulus classes (letters and nonletters show similar patterns), whereas transposition effects are amplified for letter strings. The present experiments examine, using the masked priming technique, whether the early letter-order tolerance is modulated by letter-form confusability.

The present experiments provide a direct test of whether letter-order encoding is modulated by the confusability of letter identities. If letter-order coding operates over abstract identities—as assumed by spatial coding (Davis, 2010), overlap model (Gomez et al., 2008), open-bigram models (Grainger & van Heuven, 2003; Whitney, 2001), and LCD-derived models (Dehaene et al., 2005; see Agrawal & Dehaene, 2025), then transposed-letter priming should be comparable regardless of the visual similarity of the transposed letters. If, instead, early visual-form uncertainty propagates directly into relative position coding, as may be expected under implementations of the noisy-channel model (Norris & Kinoshita, 2012), transposed-letter priming should be larger when the transposed letters are visually similar than when they are visually dissimilar.

Notably, the experiments are not intended as a test between strictly serial stage models and fully parallel models, in which partial information may become available to later computations before earlier computations reach a stable state, and factors affecting different levels may interact in some scenarios. Instead, the critical question here is whether uncertainty at the level of visual letter form propagates sufficiently to modulate the orthographic codes as indexed by masked transposed-letter priming.

We tested these predictions using uppercase primes, due to the greater number of visually confusable letter pairs in uppercase (Simpson et al., 2013). Experiment 1 served as a manipulation check. Using substitution priming, we confirmed that visual-similarity priming survives this cross-case format (e.g., *NEVTRAL*–neutral produced faster responses than *NEZTRAL*–neutral).

Experiments 2–3 provided the critical tests by contrasting transposed-letter (TL) primes with replacement-letter (RL) controls while varying the visual similarity of the transposed pair. Critically, the two target letters selected for transposition (and replacement in RL primes) were either visually similar (e.g., P and R, as in *DEPRESIÓN [depression]*) or visually dissimilar (e.g., L and S, as in EXPULSIÓN [expulsion]), allowing a direct test of whether visual similarity modulates the magnitude of transposed-letter priming. Experiment 2 used lexical decision, a task that requires lexical access. As a complement, Experiment 3 used a same–different matching task, where responses can be guided primarily by the orthographic correspondence between the reference string and the target (Norris & Kinoshita, 2008). If visual similarity modulates transposed-letter priming at an early prelexical orthographic level, the same–different matching task should provide a particularly sensitive test of this modulation.

Thus, the logic is straightforward: comparable transposed-letter priming effects for visually similar and dissimilar transpositions would support the view that order tolerance is computed over abstract identities (e.g., Davis, 2010; Dehaene et al., 2005) and would be consistent with recent neurocomputational accounts in which a later-emerging ordinal code reads out position over a representation that is not tightly coupled to letter-form confusability (Agrawal & Dehaene, 2024, 2025). In contrast, a larger transposed-letter effect for visually similar transpositions would support uncertainty-propagation accounts in which identity and order are jointly constrained early, including noisy-channel models in which identity confusability may increase the probability that a transposed input could have arisen from the target letter sequence (Norris & Kinoshita, 2012), thereby challenging the *identity-then-order* assumption.

## Experiment 1

### Method

The materials, data, code, and output of the experiment are available online (https://osf.io/2cp49/overview?view_only=782b8d95ec4b4b2891f6e575e9a218b0).

#### Participants

Thirty native speakers of Spanish (17 men, 13 women, mean age = 30.8 years, *SD* = 4.95, range: 23–40), recruited from Prolific Academic (https://www.prolific.com/), took part in the experiment and were paid at the average hourly rate on the platform. All reported normal/corrected vision and no history of reading difficulties. With 30 participants and 80 word trials in each priming condition per participant, this design yielded approximately 2,400 word observations per condition, providing a useful benchmark for stable estimation in masked priming experiments (Brysbaert & Stevens, 2018). This series of experiments was approved by the Ethics Committee of the Universitat de València, and all participants provided informed consent before the experiment.

#### Materials

The 240 target words were randomly extracted from the experiments of Marcet and Perea (2017). The mean Zipf frequency in the EsPal Subtitle database (Duchon et al., 2013) was 3.95 (range: 1.93–5.91), the mean length was 7.7 (range: 5–11), and the mean OLD20 Levenshtein distance was 2.24 (range: 1.25–4.25). Each target word (e.g., *neutral*), always presented in lowercase, was preceded by (1) an identity uppercase prime (e.g., *NEUTRAL*); (2) a visually similar prime in which an internal letter was replaced by a visually similar letter (e.g., *NEVTRAL*); or (3) a visually dissimilar prime in which an internal letter—the same as in the previous condition—was replaced by a visually dissimilar letter with the same consonant/vowel status as the visually similar letter (e.g., *NEZTRAL*). The visually similar letter replacements involved the letter pairs I and J (the similarity was 4.10 in Simpson et al., 2013, letter similarity matrix for uppercase letters) or U and V (the similarity was 5.23 in Simpson et al., 2013, matrix for uppercase letters) in either direction—for the lowercase letters these figures in the Simpson et al. (2013) matrix are 5.17 and 4.53. For the visually dissimilar letter replacements, the similarity index was less than 2.0. We also employed 240 orthographically legal pseudowords from the Marcet and Perea (2017) experiments to act as nonword targets—these had been created by Wuggy (Keuleers & Brysbaert, 2010). The manipulation for the nonword targets was the same as that of the word targets. The prime–target pairs were counterbalanced across three lists in a Latin-square manner; this way, each target appeared once per participant and occurred equally often in each priming condition.

#### Procedure

The experiment was developed in PsychoPy (Peirce et al., 2019) and deployed on Pavlovia (www.pavlovia.org). We used the standard instructions in lexical decision: participants were asked to indicate, as quickly and accurately as possible, whether the lowercase target string was a Spanish word (pressing “M”) or a nonword (pressing “Z”; see Gómez et al., 2025, for evidence that keyboard input is reliable for browser-based response time experiments). Each trial consisted of a forward mask (a row of hash marks matched in length to the target) presented for 500 ms, followed by an uppercase prime for 50 ms, and then the lowercase target, which remained on screen until response or 2,000 ms (see Angele et al., 2023, for evidence that online masked priming can reproduce laboratory masked priming effects).^1^ Target and masks were shown in a monospaced font (Courier New), and primes were rendered at 70% of target size so both the forward mask and the target effectively masked the prime. The trial order was randomized for each participant, and short breaks occurred every 80 trials. There was a 16-trial practice phase before the experimental phase. The experiment lasted approximately 20 min.

#### Data analyses

The inferential analyses used Bayesian linear mixed-effects models implemented in *brms* (Bürkner, 2017, 2018) as a front end to Stan (Stan Development Team, 2024) in R (R Core Team, 2024). For word targets, the fixed effect was Type of Prime with three levels (Identity, Visually Similar, Visually Dissimilar). We set Visually Similar as the reference level (treatment coding), which lets us estimate two planned contrasts: (1) the visual-similarity priming effect (Visually Dissimilar vs. Visually Similar) and (2) the identity advantage over visually similar primes (Visually Similar vs. Identity). The primary dependent measure was correct reaction time (RT). We modeled RTs using the ex-Gaussian distribution to capture the asymmetry of RT distributions. Accuracy was analyzed with the Bernoulli distribution. The random-effect structure was maximal: (1 + PrimeType | subject) + (1 + PrimeType | item). We used default weakly informative priors from brms. Models were run with four chains and 10,000 iterations per chain (1,000 warm-up), and chain convergence was assessed using *R*-hat. We report the medians and 95% credible intervals (CrI) of the posterior distributions of the two planned contrasts. As usual in masked priming lexical decision experiments, our only focus was on word targets, but parallel analysis was conducted with the nonword data, reported in the OSF link, and we report mean RTs and accuracy for both words and nonwords.

### Results and discussion

We excluded incorrect responses (3.3% for word trials) and very fast responses (less than 250 ms; 0 word trials [0.0%]) from the response time analyses. The mean RT and accuracy in each condition are presented in Table 1.

**Table 1:** Mean lexical decision times (in ms) and accuracy (in parentheses) for words and nonwords in Experiment 1

	Identity	Visually similar	Visually dissimilar
Words	593 (0.977)	601 (0.963)	608 (0.960)
Nonwords	656 (0.973)	652 (0.966)	655 (0.972)

Response times to the target words were faster when preceded by a visually similar prime than when preceded by a visually dissimilar prime, *b* = 8.44, Est. Error = 2.62, 95% CrI [3.30, 13.58]—note that these coefficients are model-based posterior estimates for the planned contrasts from the ex-Gaussian mixed-effects model, not raw arithmetic differences between the descriptive cell means. In addition, response times were faster when preceded by an identity prime than when preceded by a visually similar prime, *b* = −8.87, Est. Error = 3.13, 95% CrI [−15.03, −2.69]. The accuracy data did not show any credible differences, as shown in the supplementary online materials available at the OSF.

Thus, the present experiment, using uppercase primes and lowercase targets, replicated earlier experiments of visual similarity priming (e.g., Marcet & Perea, 2017): response times were faster for *NEVTRAL–neutral* than for NEZTRAL–neutral, and at the same time, we found faster responses in the identity condition (*NEUTRAL–neutral*) than in the visually similar condition. This graded pattern indicates that letter identity is encoded, but the early mapping to case-invariant letter codes remains visually tuned; hence, the advantage of visually similar over visually dissimilar primes.

Once we have shown that visual similarity priming effects can be obtained with uppercase primes and lowercase targets, the issue is whether transposed-letter priming is modulated by the visual similarity of the transposed letters.

## Experiment 2

### Method

The materials, data, code, and output of the experiment are available online (https://osf.io/2cp49/overview?view_only=782b8d95ec4b4b2891f6e575e9a218b0).

#### Participants

A new sample of 51 participants with the same characteristics as in Experiment 1 (18 men, 33 women; mean age = 30.4 years, *SD* = 4.86, range: 21–40) was recruited from Prolific. This sample size enabled more than 2,000 observations per Prime Type × Pair Similarity cell.

#### Materials

We selected 240 Spanish words from the EsPal subtitle database (Duchon et al., 2013) to serve as target words in the experiment. One hundred and twenty of these words comprised the visually similar set—they had a mean Zipf frequency of 3.8 (range: 2.1–5.4), a mean length of 8.3 (range: 6–10), and a mean OLD20 Levenshtein distance of 2.2 (range: 1.3–3.8). The other 120 words composed the set of visually dissimilar pairs—they had a mean Zipf frequency of 3.9 (range: 1.4–5.7), a mean length of 8.3 (range: 6–10), and a mean OLD20 value of 2.4 (range: 1.4–3.8). The values of these three variables were matched on the two sets of words. For each target, always presented in lowercase, we created three primes presented in uppercase: (1) an identity prime; (2) a transposed-letter prime, in which two internal letters had been transposed; or (3) a replacement-letter prime, in which the two transposed letters had been replaced by other letters matched for consonant/vowel status and selected to be minimally visually similar to the original letters (Simpson et al., 2013, similarity below 2.0). The visually similar pairs involved the letters I/T, P/R, I/L, and N/M (the letter similarity scores were 4.27, 5.27, 4.50, and 4.63, respectively, in the Simpson et al. (2013) letter similarity matrix for uppercase letters). For dissimilar pairs, scores were below 2.0, ensuring minimal visual confusability. The transpositions involved two internal adjacent letters or two internal nonadjacent letters (with one letter in between; e.g., AMINAL for ANIMAL), with the proportion for adjacent pairs being .6 in both sets of stimuli. For the two sets of words, the mean log bigram frequency was similar for the transposed-letter and the replacement-letter primes (2.0 in all cases, for the visually similar and dissimilar sets). The transposed-letter primes were constructed from either two visually similar letters (e.g., *DERPESIÓN* from the word *DEPRESIÓN*) or from two visually dissimilar letters (e.g., *EXPUSLIÓN* from the word *EXPULSIÓN*). The materials were counterbalanced across three lists such that each target word appeared in all prime conditions across participants but only once per participant. From this set, we also created 240 nonwords, using Wuggy (Keuleers & Brysbaert, 2010), with a parallel manipulation to that of the word targets.

Of note, pair similarity was manipulated between targets rather than within targets. A fully within-target manipulation would have required changing the critical letters or their positions within the same word, thereby introducing variation in local orthographic structure, letter position, and often bigram characteristics.

#### Procedure

The platform, instructions, and display settings were the same as in Experiment 1.

#### Data analyses

They were parallel to Experiment 1. We fitted a model including Prime Type factor (Identity, Transposed-Letter, Replacement-Letter) and Transposed-Pair Similarity (Similar, Dissimilar) as fixed effects. The reference level for Prime Type was the transposed-letter priming condition, allowing us to test: (1) the transposed-letter effect (i.e., replacement-letter prime vs. transposed-letter prime) and its interaction with Transposed-Pair Similarity and (2) the advantage of the identity prime over the transposed-letter prime (i.e., transposed-letter prime vs. identity prime) and its interaction with Transposed-Pair Similarity. We used the maximal random-effects structure: (1 + PrimeType × PairSimilarity | subject) + (1 + PrimeType | item). The analyses on the nonword targets are provided in the OSF.

### Results and discussion

We removed the incorrect responses (4.5% for word trials) and very fast responses (less than 250 ms; 2 word trials [<0.1%]) from the response time analyses. The mean RT and accuracy per condition are displayed in Table 2.

**Table 2:** Mean lexical decision times (in ms) and accuracy (in parentheses) for words and nonwords in Experiment 2

	Identity prime	Transposed-letter prime	Replacement-letter prime
*Word trials—Transposed-Pair Similarity*			
Visually Similar	617 (0.962)	621 (0.963)	636 (0.950)
Visually Dissimilar	614 (0.960)	618 (0.950)	628 (0.945)
*Nonword trials— Transposed-Pair Similarity*			
Visually Similar	683 (0.965)	679 (0.969)	674 (0.964)
Visually Dissimilar	692 (0.952)	686 (0.959)	687 (0.949)

**Fig. 1:**
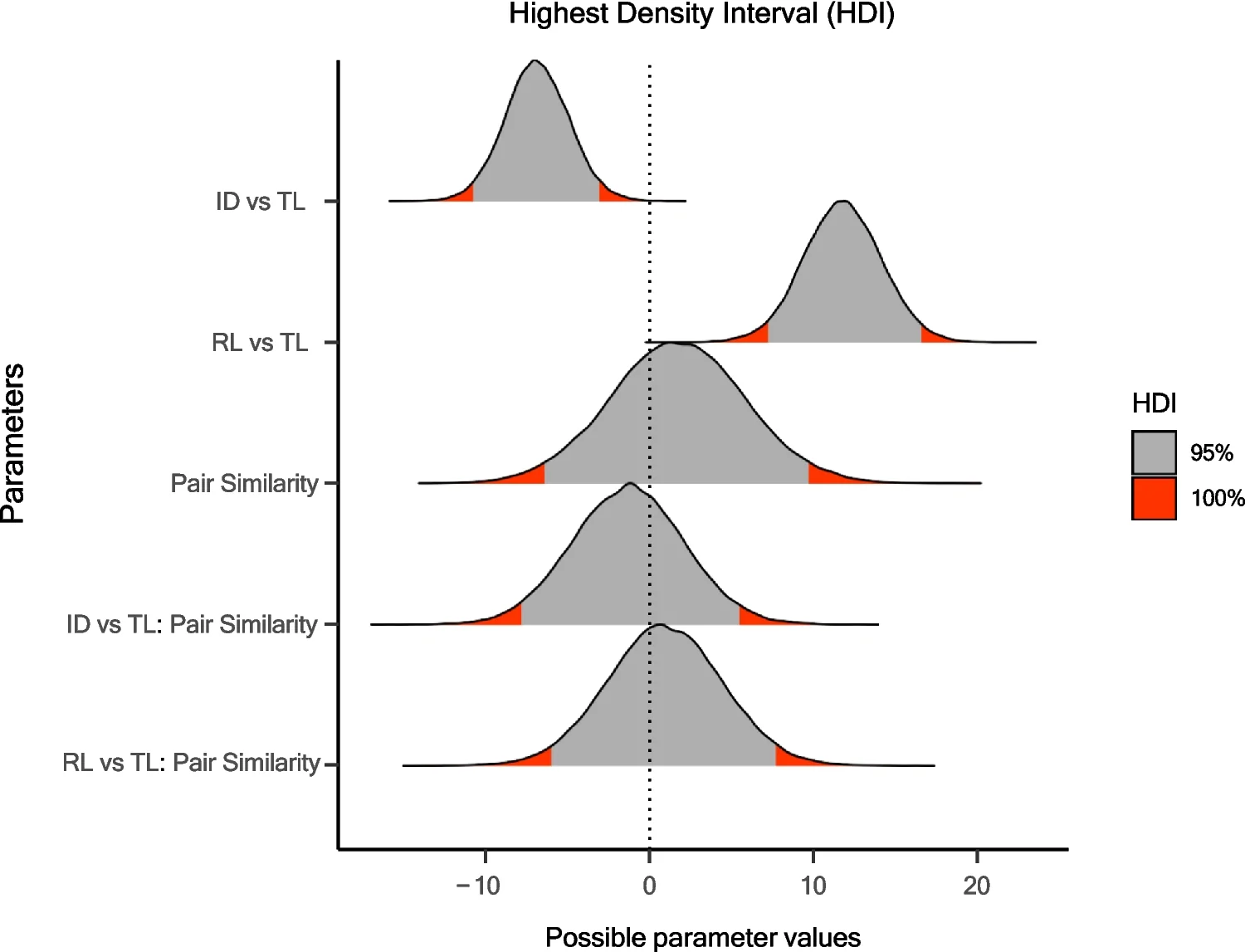
Posterior distributions of the fixed-effect estimates from the Bayesian linear mixed-effects model fitted to correct lexical decision times (Experiment 2; word trials). Prime type was treatment-coded with TL (transposed-letter prime) as the reference level. The parameters were ID vs. TL, RL vs. TL, Pair Similarity, (ID vs. TL) × Pair Similarity, and (RL vs. TL) × Pair Similarity. Grey shading denotes the 95% credible interval for each posterior distribution. (Color figure online)

We found faster response times to target words when preceded by a transposed-letter prime than when preceded by a replacement-letter prime, *b* = 11.75, Est. Error = 2.39, 95% CrI [7.05, 16.43], and when preceded by an identity prime than when preceded by a transposed-letter prime, *b* = −6.86, Est. Error = 1.96, 95% CrI [−10.70, −2.97]—as in Experiment 1, these coefficients are model-based posterior estimates from the model, not raw arithmetic differences between the descriptive cell means. Critically, neither of these effects was modulated by visual similarity (RL–TL × Pair Similarity interaction: *b* = 0.92, Est. Error = 3.50, 95% CrI [−5.91, 7.76]; ID-TL × Pair Similarity interaction: *b* = −1.30, Est. Error = 3.50, 95% CrI [−7.90, 5.40]) (see Fig. 1. for the posterior distributions)

Thus, the present lexical decision experiment showed a transposed-letter priming effect that was remarkably similar regardless of the visual similarity of the transposed letters. The question now is whether this modulation (if any) can be found in an orthographic same-different matching task.

## Experiment 3 (same-different matching task)

### Method

The materials, data, code, and output of the experiment are available online (https://osf.io/2cp49/overview?view_only=782b8d95ec4b4b2891f6e575e9a218b0).

#### Participants

We recruited a new sample of 51 participants, with the same characteristics as in Experiment 2 (19 men, 31 women, one other; mean age = 29.9 years, *SD* = 5.05, range: 21–40).

#### Materials

The 240 target words and their corresponding primes were the same as in Experiment 2, and they served as “same” reference-target trials in the experiment. For the “different” reference-target trials, we selected a new set of 240 target words from the EsPal database (Duchon et al., 2013); for these words, the mean Zipf frequency was 4.3 (range: 3.4–5.5), the mean length was 8.3 (range: 6–10), and the mean OLD20 was 2.2 (range: 1.3–3.9)—the “reference” words for these target stimuli had a mean Zipf frequency of 4.3 (range: 3.4–5.5), a mean length was 8.3 (range: 6–10), and a mean OLD20 of 2.2 (range: 1.1–3.9). For the “different” trials, the prime (identity, transposed-letter, replacement-letter) was related to the reference rather than to the target (i.e., zero-contingency scenario). Given that there was no prime/target manipulation for “different” trials, the transposed-letter primes did not involve a manipulation of visual similarity.

#### Procedure

The general details were similar to those of Experiment 2, except for two key details. First, the instructions were for a same–different matching task. Participants were asked to indicate whether two stimuli (one in uppercase and the other in lowercase) presented consecutively on the screen were the same letter string (ignoring letter case), pressing “M” (same) or not, pressing “Z” (different). Second, the trial began with an uppercase reference word centered slightly above fixation for 300 ms. While the reference was on screen, a forward mask (a series of #s matched in length to the target) occupied the central fixation region. Immediately after the reference offset, the mask was replaced by a briefly presented uppercase prime word (50 ms) that was 70% of the font size of the target. The prime was followed by a lowercase target word, which remained visible until response or a 2,000-ms deadline.

#### Data analyses

They were parallel to those in Experiment 2—note that the focus was on “same” trials.

### Results and discussion

We excluded error responses (3.2% for “same” trials) and very fast responses (less than 250 ms; 1 “same” trial [<0.1%]) from the RT analyses. The mean response times and accuracy in each condition for “same” trials are displayed in Table 3.

**Table 3:** Mean same-different matching times (in ms) and accuracy (in parentheses) for words in Experiment 3

	Identity prime	Transposed-letter prime	Replacement-letter prime
Visually similar	502 (0.972)	506 (0.975)	526 (0.950)
Visually dissimilar	497 (0.972)	502 (0.974)	524 (0.965)

The mean correct RTs and accuracy for “different” trials were 542 ms and 0.981, respectively

We found faster response times to target words when preceded by a transposed-letter prime than when preceded by a replacement-letter prime, *b* = 18.17, Est. Error = 1.99, 95% CrI [14.26, 22.09], and when preceded by an identity prime than when preceded by a transposed-letter prime, *b* = −3.67, Est. Error = 1.61, 95% CrI [−6.87, −0.52]. As in the lexical decision task, we found no signs that this pattern was affected by the visual similarity of the transposed letters (RL–TL × Pair Similarity interaction, *b* = −1.29, Est. Error = 3.13, 95% CrI [−7.45, 4.86]; ID vs. TL × Pair Similarity interaction, *b* = −0.82, Est. Error = 2.94, 95% CrI [−6.55, 4.97]).

**Fig. 2:**
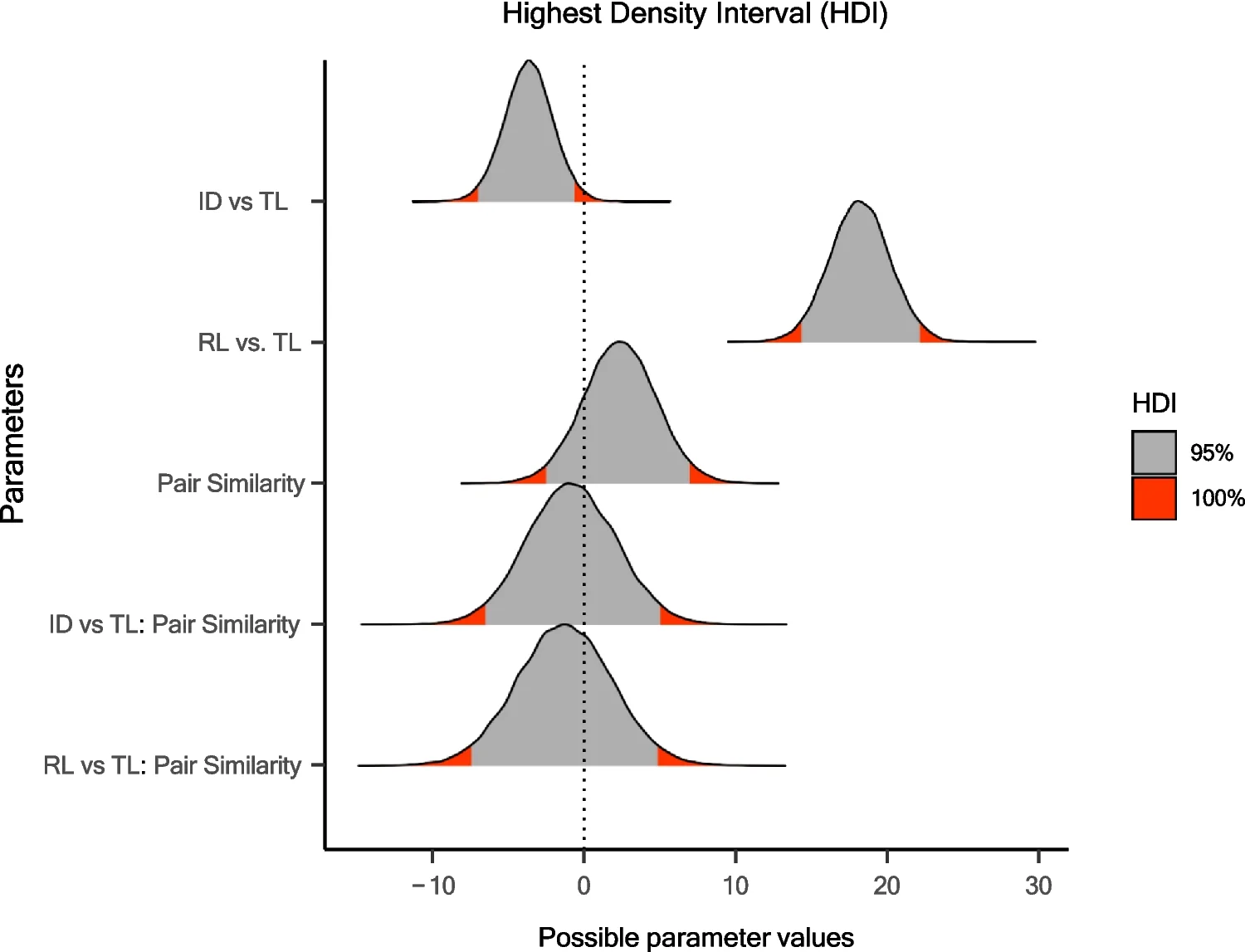
Posterior distributions of the fixed-effect estimates from the Bayesian linear mixed-effects model fitted to correct response times in the same-different task (Experiment 3; “same” trials). The coding was the same as in Experiment 2. Grey shading denotes the 95% credible interval for each posterior distribution. (Color figure online)

Again, the posterior distribution for the critical interaction was centered near zero, providing little evidence for a modulation of transposed-letter priming by visual similarity (see Fig. 2). Thus, the same-different experiment converged with the lexical decision experiment: robust TL priming was observed, but it was not detectably modulated by the visual similarity of the transposed letters.

## General discussion

The present experiments examined whether the visual similarity of transposed letters modulates transposed-letter priming, a key marker of flexible letter-order coding. We contrasted TL primes involving visually similar pairs (e.g., P↔R; DERPESIÓN–depresión) with TL primes involving visually dissimilar pairs (e.g., L↔S; EXPUSLIÓN–expulsión). In both lexical decision and same–different matching experiments, transposed-letter priming was sizable and of comparable magnitude for visually similar and visually dissimilar transpositions. Importantly, Experiment 1 showed reliable visual-similarity priming effects with uppercase primes and lowercase targets (e.g., NEVTRAL–neutral produced faster responses than NEZTRAL–neutral; Perea et al., 2008; see also Kinoshita et al., 2014), confirming that the visual-form manipulation was effective under the present setup. Thus, the critical empirical pattern is clear: visual similarity affected substitution priming, whereas masked transposed-letter priming remained stable across visually similar and visually dissimilar transpositions.

Although null interactions require caution, the Bayesian analyses yielded interaction estimates centered close to zero in both tasks: lexical decision, *b* = 0.92 ms, 95% CrI [–5.91, 7.76]; same–different matching, *b* = –1.29 ms, 95% CrI [–7.45, 4.86]. The posterior mass also supported a practically negligible interaction, with p(|interaction| < 5 ms) = .834 in lexical decision and .861 in same–different; p(|interaction| < 10 ms) = .994 and .996, respectively (see OSF for further details). Thus, while very small modulations, of the order of a few milliseconds, remain possible, the present data provide little support for a sizeable modulation of transposed-letter priming by visual similarity. At the same time, because behavioral response times provide an endpoint measure of processing, the present experiments cannot exclude the possibility that visual letter form produced a short-lived modulation at an earlier stage, before the emergence of the more abstract orthographic code reflected in transposed-letter priming effects. This possibility is motivated by previous masked priming event-related potentials (ERP) experiments showing that visual-format manipulations (e.g., changes in font or letter case) can modulate early activity in the N/P150 range while leaving later orthographic/lexical components—and, in some cases, behavioral measures—largely unaffected (e.g., see Chauncey et al., 2008; Vergara-Martínez et al., 2015).^2^ Future research using ERPs would provide a more direct test of whether visual similarity produces such a transient early modulation in the encoding of letter order, even when it leaves no measurable behavioral trace.

The absence of a detectable modulation of transposed-letter priming by visual similarity is most naturally accommodated by models in which flexible letter-order coding operates over abstract letter identities (Davis, 2010; Dehaene et al., 2005; Gómez et al., 2008; Grainger & van Heuven, 2003; Grainger & Ziegler, 2011; Snell, 2025; Whitney, 2001). For example, in the Local Combination Detector model (Dehaene et al., 2005), visual features feed case-invariant letter representations, which in turn feed ordered multi-letter codes. The present data are consistent with this class of identity-first implementations: a manipulation that affected letter-form processing in Experiment 1 did not measurably modulate the transposed-letter priming effect in Experiments 2–3. A similar interpretation applies to PONG’s identity-first activation and bihemispheric estimation of letter order (Snell, 2025), in which coarse order is estimated over abstracted letter identities rather than over low-level visual forms (see Snell et al., 2023, for discussion). Conversely, models in which visual-form uncertainty is expected to feed directly into position uncertainty would predict a larger transposed-letter advantage for visually similar transpositions than for visually dissimilar transpositions. This was not observed. Thus, while the present findings do not falsify noisy-channel processing in general, they constrain implementations in which uncertainty about letter identity and uncertainty about letter position are assumed to be tightly coupled during the computations that give rise to masked transposed-letter priming effects.

The same conclusion is compatible with recent neurocomputational models in which retinotopic letter coding is progressively transformed into an ordinal code anchored to word boundaries (Agrawal & Dehaene, 2024, 2025), with ordinal coding emerging only after the initial retinotopic coding phase, while retinotopic information remains available. From this perspective, the present findings add an important behavioral constraint: a manipulation that affects letter-form processing, as shown by visual-similarity priming in Experiment 1, did not measurably propagate into the mechanism responsible for masked transposed-letter priming in Experiments 2–3.

The present dissociation also dovetails with the unprimed same–different matching results reported by Massol and Grainger (2024), where visual-similarity effects generalized across letters and nonletters, whereas transposition effects were amplified for letter strings. The present findings extend this dissociation to masked priming: even when visual-form similarity was manipulated within the transposed pair itself, it did not increase transposed-letter priming. Critically, this pattern was observed not only in lexical decision but also in same–different matching, a task that provides a sensitive measure of early orthographic overlap while reducing the contribution of lexical selection processes (Norris & Kinoshita, 2008). The convergence across tasks strengthens the claim that visual-form similarity and letter-order flexibility make separable contributions to early orthographic processing. This pattern also complements recent parafoveal evidence showing that letter-order sensitivity is shaped by eccentricity, visual field, and the position of the transposed letters within the string (Veldre et al., 2024). Taken together, these findings point to letter-order coding as a relational process that is sensitive to spatial constraints but not necessarily to the visual similarity of the letters whose order is being encoded.

In sum, visual similarity influenced substitution priming in Experiment 1 with uppercase primes and lowercase targets, confirming that the manipulation affected early letter-form processing, but it did not produce a detectable modulation of transposed-letter priming in either lexical decision or same–different matching. This pattern supports the core assumption of identity-then-order accounts: the flexible order code responsible for transposed-letter priming operates over letter identities that are already substantially abstracted from visual form. More generally, these findings place quantitative constraints on models in which uncertainty about letter identity is expected to propagate into uncertainty about letter order. A comparable tolerance for order has also been found at the word level, where readers can efficiently process sentences containing adjacent-word transpositions (transposed-word effect; Mirault et al., 2018), suggesting that order assignment may operate over already-identified representations at multiple levels of the reading system.

## Data Availability

The data and materials of the three experiments are available online (https://osf.io/2cp49/overview?view_only=782b8d95ec4b4b2891f6e575e9a218b0).

## Footnotes

1 Although web-based experimental software has demonstrated strong capabilities for stimulus presentation and online masked priming experiments have repeatedly reported effects parallel to those obtained in the laboratory (e.g., see Angele et al., 2023, for discussion), we acknowledge that some caution is necessary because there is no full control over participants’ computer displays; nonetheless, as Forster et al. (2003) noted, with prime durations in the 30–50-ms range, the very short prime–target stimulus-onset asynchrony makes strategic or retroactive use of the prime highly unlikely, even when some residual prime information is available.

2 This qualification is also relevant to letter-case information. During reading, letter case may remain available when it serves higher-level functions (e.g., capitalization or proper-name marking; see Labusch et al., 2022; Peressotti et al., 2003).

## Code availability

The code of the three experiments is available online (https://osf.io/2cp49/overview?view_only=782b8d95ec4b4b2891f6e575e9a218b0).
